# Risk factors for intraventricular hemorrhage in very low birth weight infants: a systematic review and meta-analysis

**DOI:** 10.3389/fped.2025.1728632

**Published:** 2026-01-26

**Authors:** Liming Bao, Jinyan Huang

**Affiliations:** The Second Affiliated Hospital of Wenzhou Medical University, Zhejiang, China

**Keywords:** intraventricular hemorrhage, meta-analysis, risk factors, systematic review, very low birth weight infant

## Abstract

**Background:**

Intraventricular Hemorrhage (IVH) is one of the common and serious complications of Very Low Birth Weight Infant (VLBW) that may lead to long-term neurodevelopmental deficits. Although several studies have been conducted to explore its risk factors, the results have been inconsistent. The aim of this study was to identify the major risk factors for intraventricular Hemorrhage in VLBW by systematic evaluation and Meta-analysis of the available evidence.

**Methods:**

PubMed, Web of Science, Embase, Cochrane Library were systematically searched, and observational studies (case-control and cohort studies) were included from the time of library construction to 20 January 2025, and the literature that met the criteria were screened and relevant data were extracted. Meta-analysis was performed using Stata 15.0 software to assess the combined odds ratio (OR) and 95% confidence interval (CI) for each risk factor.

**Results:**

A total of 21 studies included 6 case-control studies, 15 cohort studies, involving a total of 13,800 patients, The results of the meta-analysis showed that hypotension [OR = 3.64, 95%CI (1.87, 7.08)], patent ductus arteriosus (PDA) [OR = 1.86, 95%CI (1.46, 2.36)], vaginal delivery [OR = .10, 95%CI (1.61, 2.72)], neonatal thrombocytopenia[OR = 2.43, 95%CI (1.79, 3.30)], pulmonary hemorrhage [OR = 2.45, 95%CI (1.43, 4.20)], mechanical [OR = 2.09, 95%CI (1.40, 3.10)], sepsis[OR = 2.28, 95%CI (1.77, 2.95)] were a risk factor for the development of IVH in VLBW. While antenatal corticosteroids [OR = 0.68, 95%CI (0.55, 0.84)] was a protective factor for the development of IVH in VLBW.

**Conclusion:**

This study indicates that hypotension, patent ductus arteriosus (PDA), antenatal corticosteroid use, vaginal delivery, neonatal thrombocytopenia, pulmonary hemorrhage, mechanical ventilation, and sepsis constitute the primary risk factors for IVH in VLBW infants. Although these factors exhibit strong clinical associations, current understanding of IVH pathogenesis remains largely dependent on preclinical studies. Integrating clinical and preclinical evidence facilitates a more comprehensive understanding of IVH etiology and informs early intervention strategies.

**Systematic Review Registration:**

identifier CRD42025633474.

## Background

With the continuous improvement of perinatal medicine, prenatal monitoring technology and neonatal intensive care, the survival rate of preterm infants has significantly improved, especially Very Low Birth Weight Infant (VLBW), newborns with a birth weight of less than 1,500 grams, whose survival rates have improved markedly, rising from about 66.8% in the mid-1990s (1994–1998) to around 90.0% in the mid-2010s (2014–2019) in cohort studies at tertiary centers, reflecting an approximate 23 percentage point increase over ∼25 years (1, 2). However, due to the immaturity of their physiological structures and functions, these infants are still at risk of many serious complications, of which IVH is one of the most common and potentially disabling (3). Specifically, we now state that approximately 20%–40% of children with severe IVH develop cerebral palsy, 30%–50% experience cognitive impairment, 20%–35% develop post-hemorrhagic hydrocephalus, and 5%–10% develop epilepsy. IVH not only affects the mortality rate in the neonatal period, but is also associated with neurological delay, cerebral palsy, cognitive impairment, and another long-term prognosis (4, 5).

According to Papile's classification (6), IVH can be classified into grades I–IV, with grade II being periventricular hemorrhage, grade II being entry into the ventricles without ventricular dilatation, grade III being intraventricular Hemorrhage with ventricular dilatation, and grade IV being intraventricular Hemorrhage with parenchymal hemorrhage (7, 8). Higher grades of IVH often indicate more serious conditions and worse prognosis. According to relevant studies, the incidence of IVH in VLBW can be as high as 25% to 50%, with severe IVH (grade IIIIV) accounting for about 8% to 15% (9, 10). Because of the immature neurodevelopment of these infants, the thin wall of cerebral blood vessels, and the poor self-regulation ability of the brain, they are very prone to IVH under the combined effect of many internal and external factors (11).

The mechanism of IVH is a complex process with the interaction of many factors. In VLBW, the vessel wall of the germinal matrix, an embryonic structure in the brain, is weak and prone to rupture, and the cerebral blood flow is poorly regulated, which may be easily triggered by hemodynamic fluctuations (12), such as the fluctuation of blood pressure caused by rapid infusion of fluids and mechanical ventilation, or acidosis and hypoxia. In addition, perinatal hypoxia, infection, and coagulation disorders may also increase the risk of IVH (13). In addition, perinatal hypoxia, infections, and coagulation disorders also increase the risk of IVH. Therefore, an in-depth investigation of the risk factors of IVH is important for the development of effective early intervention and prevention strategies to improve the quality of neonatal survival (14).

Despite multiple observational studies (15–18) in recent years exploring potential risk factors for IVH in VLBW infants, existing evidence remains markedly inconsistent. Discrepancies in research design, sample size, regional population variations, and statistical methodologies have led to divergent findings, with some conclusions even proving contradictory. Consequently, there is an urgent need for a comprehensive and quantitative assessment of these risk factors to provide clearer clinical guidance (19). This study aims to address this research gap by systematically retrieving observational studies concerning risk factors for i IVH in VLBW infants. Through meta-analysis, it will synthesize the effect sizes of relevant variables, thereby providing evidence-based support for clinicians to optimize perinatal and neonatal management strategies. Ultimately, the study seeks to reduce the incidence of IVH and improve the long-term neurodevelopmental outcomes of preterm infants.

## Methods

This systematic evaluation and meta-analysis strictly followed the PRISMA (Preferred Reporting Items for Systematic Reviews and Meta-Analyses) guidelines (20). The systemic review was supported by the online PROSPERO international prospective register of systemic reviews of the National Institute for Health Research, registration number: (CRD42025633474).

### Inclusion and exclusion criteria

#### Inclusion criteria

The literature included in this study consisted of observational studies, such as case–control and cohort studies, conducted in very low birth weight infants (VLBW; birth weight <1,500 g). The included studies were required to provide a clear diagnosis of intraventricular hemorrhage (IVH) and to grade IVH using the Papile grading system to ensure the reliability of the results. In addition, the assessment of risk factors had to be clearly described, and the data had to be sufficiently complete to support valid statistical analyses.

#### Exclusion criteria

Studies were excluded if they involved infants who were not very low birth weight (i.e., birth weight ≥1,500 g), were not preterm, or had a different clinical background. Studies without a definitive diagnosis of IVH or those that did not use a standardized grading system for IVH (Papile grading) were also excluded. Furthermore, studies with incomplete data or those from which critical data could not be extracted were excluded, as were non-original research articles, including case reports, case series, review articles, and laboratory-based studies.

### Literature search

A systematic search of PubMed, Web of Science, Embase, and the Cochrane Library was performed to include observational studies (case-control and cohort studies) from the time of their construction to 20 January 2025. The search terms were (Infant, Very Low Birth Weight [MeSH Terms] OR Infant, Very Low Birth Weight [Title/Abstract] OR Very-Low-Birth-Weight Infant [Title/Abstract]) AND (Cerebral Intraventricular Hemorrhage [MeSH Terms] OR Cerebral Intraventricular Hemorrhage [Title/Abstract] OR (Cerebral Intraventricular Hemorrhages [Title/Abstract] OR (Hemorrhage, Cerebral Intraventricular [Title/Abstract]) AND (Risk Factors [MeSH Terms] OR Risk Factors [Title/Abstract]) OR Factor, Risk [Title/Abstract] OR (Risk Factor [Title/Abstract]); and the specific search strategy is described in Supplementary Table S1.

### Study selection

During the literature screening process, two researchers independently used EndNote 21 software to initially screen the literature obtained from the search, first through the titles and abstracts, and then to exclude literature that clearly did not meet the inclusion criteria. Subsequently, the remaining literature was reviewed by reading the full text in its entirety to further determine whether it met the inclusion and exclusion criteria. In case of disagreement between the two researchers during the screening process, it would be resolved through discussion and negotiation; if the negotiation still failed to reach a consensus, a third researcher would be invited to adjudicate to ensure the objectivity and consistency of the screening process.

### Data extractions

This study was conducted by two researchers who independently extracted relevant data from the eligible literature using an Excel sheet based on the inclusion criteria. The extraction included the basic information of the study (first author, year of publication, country and type of study), the basic characteristics of the study population (sample size, gender, and gestational age), the statistical model used in the regression analysis, and the effect sizes of the exposure factors and the outcome indexes (the ratio than the ORs and their 95% confidence intervals). In the process of data extraction, if two investigators disagreed on the data, it would be resolved through negotiation, to ensure the accuracy and consistency of data extraction. For studies with missing data, we first contact the authors. If the data can be obtained, the study is included; otherwise, it is excluded.

### Quality evaluation

Quality will be evaluated using the NOS score (Newcastle-Ottawa Scale) (21). This scoring system rates the quality of a study by assessing three key aspects of the study: selection bias, comparability of comparison groups (comparability) and assessment of outcomes (results). In terms of selection bias, it assesses how the study selected the study population, including whether the inclusion criteria were clearly defined and whether randomized or systematic methods were used to select the study population; in terms of comparability of the comparison group, it assesses the comparability of the control group with the experimental group in terms of the key characteristics (age, sex, BMI) and examines whether potential confounders have been controlled for; and in terms of assessment of outcome, it assesses the reliability of the outcome by including the definition of the outcome, the method of measurement, and the completeness of follow-up. The quality of the study will be assessed by assigning scores to each aspect according to the specifics of the study, resulting in an overall score. The results of the scoring will help to identify low-quality studies, thus ensuring the reliability of the Meta-analysis results.

### Statistical analysis

In this study, the risk ratio (OR) and the corresponding 95% confidence interval (CI) of each included study were combined using Stata 15 software. First, for each study, we extracted the corresponding effect size OR and its 95% confidence interval. To combine these ORs, we pooled them using a random effects model, which can account for heterogeneity between studies, i.e., variability in effect sizes across studies. ORs and 95% CIs were calculated for each study and combined into an overall effect size. Heterogeneity of the model was assessed by the *I*^2^ statistic; if the *I*^2^ was greater than 50%, it was considered that there was a high degree of heterogeneity and that the sources of heterogeneity needed to be further explored. For high heterogeneity, we may conduct sensitivity analyses to identify potential factors that may affect the combined effect sizes. In addition, funnel plots and Egger's test were used to assess the likelihood of publication bias. If bias exists, it may have an impact on the interpretation of the results. The combined effect sizes will be reported as ORs and their 95% CIs to allow for interpretation of results and statistical inference. If *P* < 0.05, the results are statistically significant.

## Results

### Meta-analysis screening results

**Figure 1:**
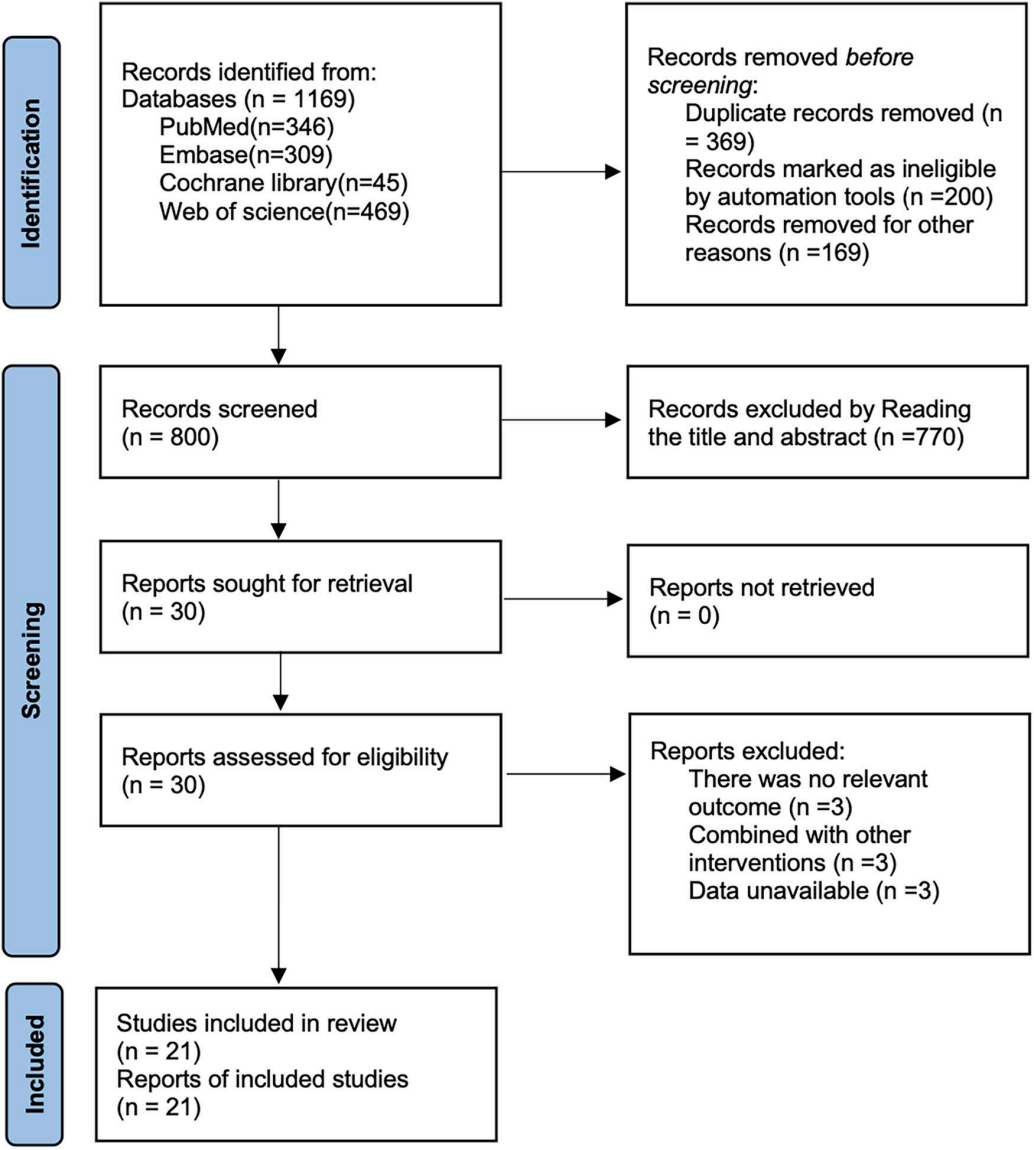
Literature search flow chart.

A total of (*n* = 1,169 studies) were retrieved by searching PubMed (*n* = 346 studies), Embase (*n* = 309 studies), Cochrane library (*n* = 45 studies), and Web of science (*n* = 469 studies) by removing duplicates (*n* = 369 studies), removing literature by reading titles and abstracts (*n* = 770 studies), and by reading full text removal of literature (*n* = 9 studies), and finally 21 articles (22–42) were included, as shown in the search flowchart in Figure 1.

### Basic characteristics of the included literature

A total of 21 studies included 6 case-control studies, 15 cohort studies, involving a total of 13,800 patients, and among these studies, there were a total of 7 studies in the United States and 4 studies in Germany. Other countries had relatively fewer studies, such as China and Israel with 2 studies each, and Poland, Iran, Turkey, Saudi Arabia, Czech Republic, Italy, and Nigeria with 1 study. And the gestational age of most of the studies was between 25 and 30 weeks, all studies used logistic regression model for data analysis and the basic characteristics are shown in Table 1.

**Table 1:** Table of basic characteristics of the literature.

Study	Year	Country	Study design	Sample size	Gender (M/F)	Gestational age (week)	Birth weight (g)	Regression model
Puerta-Martínez (36)	2024	Germany	Case–control study	90	48/44	29.1	1,034	Logistic
Humberg (28)	2017	Germany	Cohort study	2,203	1,260/843	27	899	Logistic
Vogtmann (40)	2021	Germany	Case–control study	1,782	917/865	28	<1,500	Logistic
Sauer (39)	2016	America	Cohort study	308	169/139	26	<1,500	Logistic
Kahn (30)	2002	America	Cohort study	1,283	668/615	27	<1,500	Logistic
Helwich (27)	2017	Polska	Cohort study	936	540/396	28	<1,500	Logistic
Khodapanahandeh (31)	2008	Iran	Case–control study	121	56/65	30	1,065	Logistic
Jen (29)	2006	America	Case–control study	200	98/102	25	1,239	Logistic
Ongun (34)	2021	Turkey	Cohort study	455	224/231	28	982	Logistic
Bermick (25)	2016	America	Cohort study	216	111/105	25	1,056	Logistic
Roberts (37)	2018	America	Cohort study	288	135/153	25	<1,500	Logistic
Roberts (38)	2022	America	Cohort study	120	60/60	27	<1,500	Logistic
Farghaly (26)	2024	America	Cohort study	2,895	1,476/1,419	28	<1,500	Logistic
Al-Mouqdad (23)	2021	Saudi Arabia	Case–control study	216	111/105	26	<1,500	Logistic
Linder (33)	2003	Israel	Case–control study	105	61/44	29	1,023	Logistic
Korcek (32)	2024	Czech Republic	Cohort study	1,279	712/567	27	1,089	Logistic
Parodi (35)	2020	Italy	Cohort study	286	135/151	28	900	Logistic
Adegoke (22)	2014	Nigeria	Cohort study	87	39/48	30	<1,500	Logistic
Audeh (24)	2011	Israel	Cohort study	271	149/122	29	<1,500	Logistic
Xing (41)	2022	China	Cohort study	238	146/92	27	<1,500	Logistic
Zhao (42)	2022	China	Cohort study	421	204/217	28	<1,500	Logistic

### Quality assessment results

Table 2 demonstrates the quality scores of different case-control studies and cohort studies. Most of the case-control studies scored between 8 and 9, showing high quality, with C. Vogtmann (40), Fariba Khodapanahandeh (31), and H. C. Jen (29) all scoring the highest 9. The cohort studies also scored relatively well, with most of the studies scoring between 8 and 9, except for J. C. Roberts (38), which scored slightly lower (7), indicating that these cohort studies generally had good design and analytic quality. Overall, the quality of these studies was high.

**Table 2:** NOS score results.

Study	Is the case definition adequate?	Representativeness of the cases	Determination of control group	Definition of Controls	Comparability of cases and controls based on the design or analysis	Ascertainment of exposure	Same method of ascertainment for cases and controls	Nonresponse	Total scores
case control									
Puerta-Martínez (36)	*	*	*	*	*	*	*	*	8
Vogtmann (40)	*	*	*	*	**	*	*	*	9
Khodapanahandeh (31)	*	*	*	*	**	*	*	*	9
Jen (29)	*	*	*	*	**	*	*	*	9
Al-Mouqdad (23)	*	*	*	*	*	*	*	*	8
Linder (33)	*	*	*	*	*	*	*	*	8
Study	Representativeness of the exposed group	Selection of non-exposed groups	Determination of exposure factors	Identification of outcome indicators not yet to be observed at study entry	Comparability of exposed and unexposed groups considered in design and statistical analysis	Design and statistical analysis	Adequacy of the study's evaluation of the outcome	Adequacy of follow-up in exposed and unexposed groups	Total scores
cohort study									
Humberg (28)	*	*	*	*	**	*	*	*	9
Sauer (39)	*	*	*	*	*	*	*	*	8
Kahn (30)	*	*	*	*	**	*	*	*	9
Helwich (27)	*	*	*	*	**	*	*	*	9
Ongun (34)	*	*	*	*	*	*	*	*	8
Bermick (25)	*	*	*	*	*	*	*	*	8
Roberts (37)	*	*	*	*	*	*	*	*	8
Roberts (38)	*	*	*	/	*	*	*	*	7
Farghaly (26)	*	*	*	*	*	*	*	*	8
Korcek (32)	*	*	*	*	**	*	*	*	9
Parodi (35)	*	*	*	*	**	*	*	*	9
Adegoke (22)	*	*	*	*	*	*	*	*	8
Audeh (24)	*	*	*	*	*	*	*	*	8
Xing (41)	*	*	*	*	*	*	*	*	8
Zhao (42)	*	*	*	*	*	*	*	*	8

### Meta analysis results

#### Hypotension

**Figure 2:**
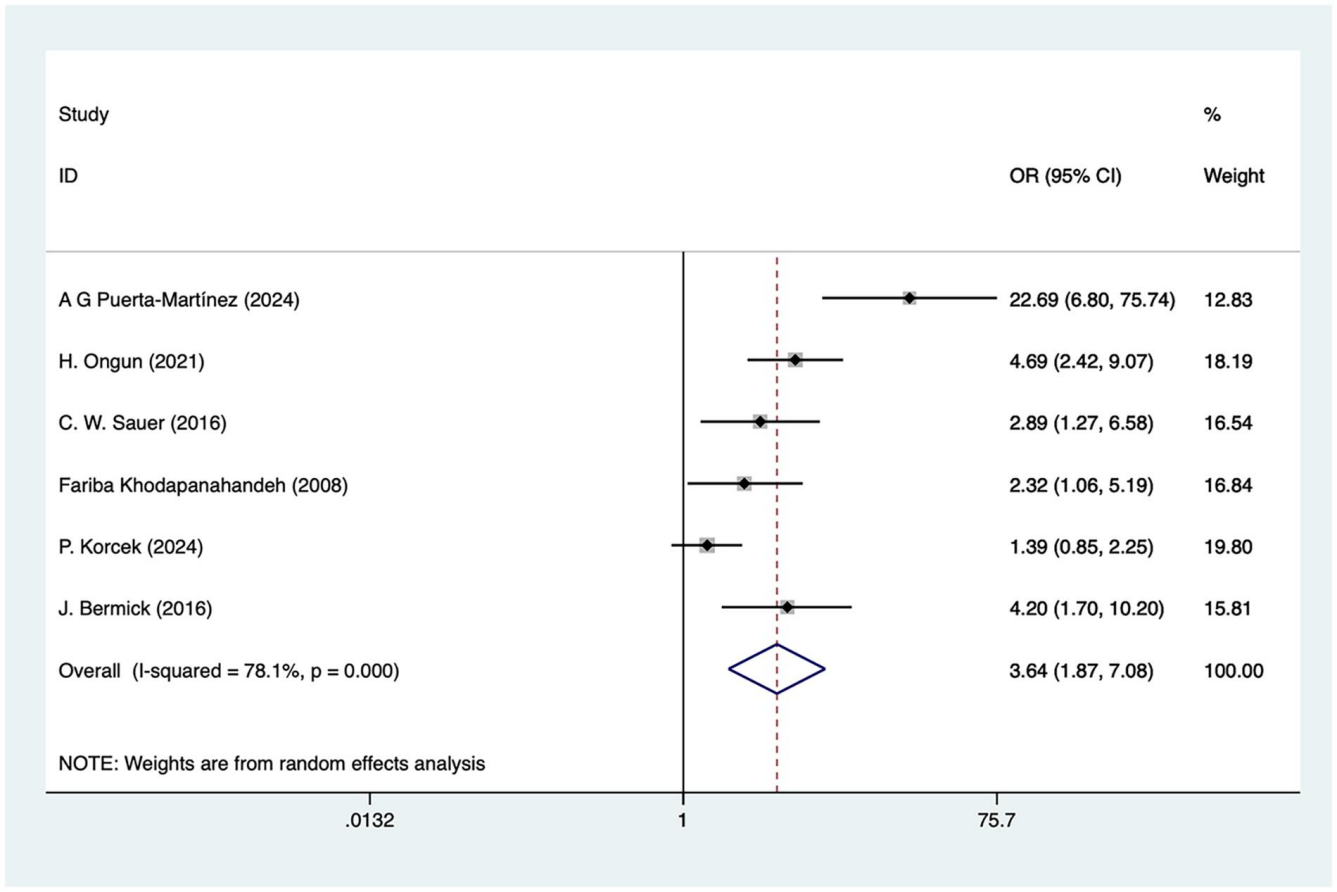
Forest plot of hypotension meta-analysis.

Six studies mentioned hypotension, and the test of heterogeneity (*I*^2^ = 78.1%, *P* = 0.001) was analyzed using a random-effects model, and the results of the analyses (Figure 2) suggested that hypotension was a risk factor for the development of IVH in VLBW [OR = 3.64, 95%CI (1.87, 7.08)]. Due to the high heterogeneity, sensitivity analysis was performed using literature-by-exclusion, and the results of the analysis (Supplementary Figure S1) suggested that the sensitivity was low and that the results of the analysis were not affected by an individual study.

#### Patent ductus arteriosus

**Figure 3:**
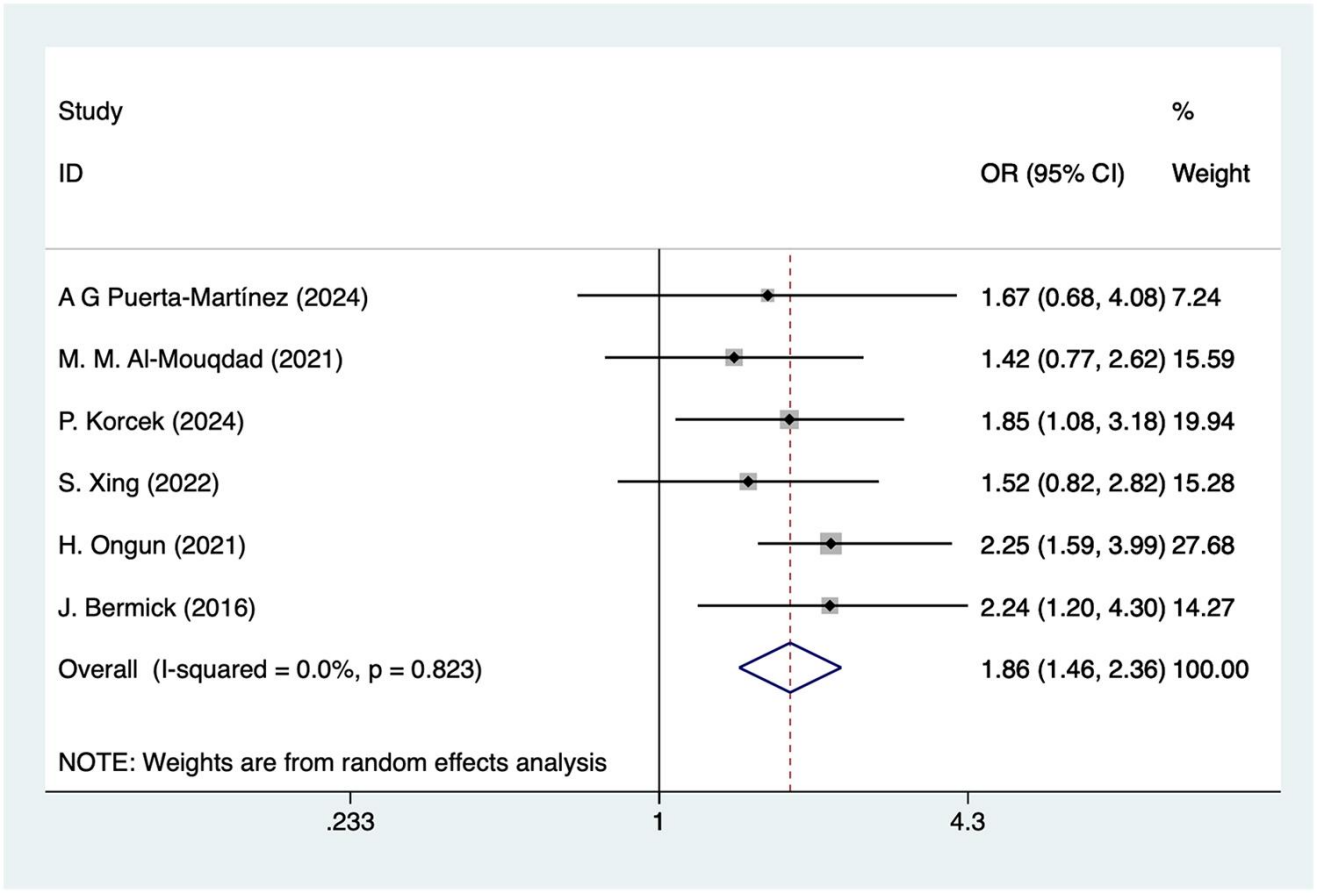
Forest plot of patent ductus arteriosus meta-analysis.

Six studies mentioned patent ductus arteriosus (PDA), and the pooled results (Figure 3) indicated that PDA was associated with an increased risk of IVH in VLBW infants [OR = 1.86, 95%CI (1.46, 2.36), *P* = 0.001].

#### Antenatal corticosteroids

**Figure 4:**
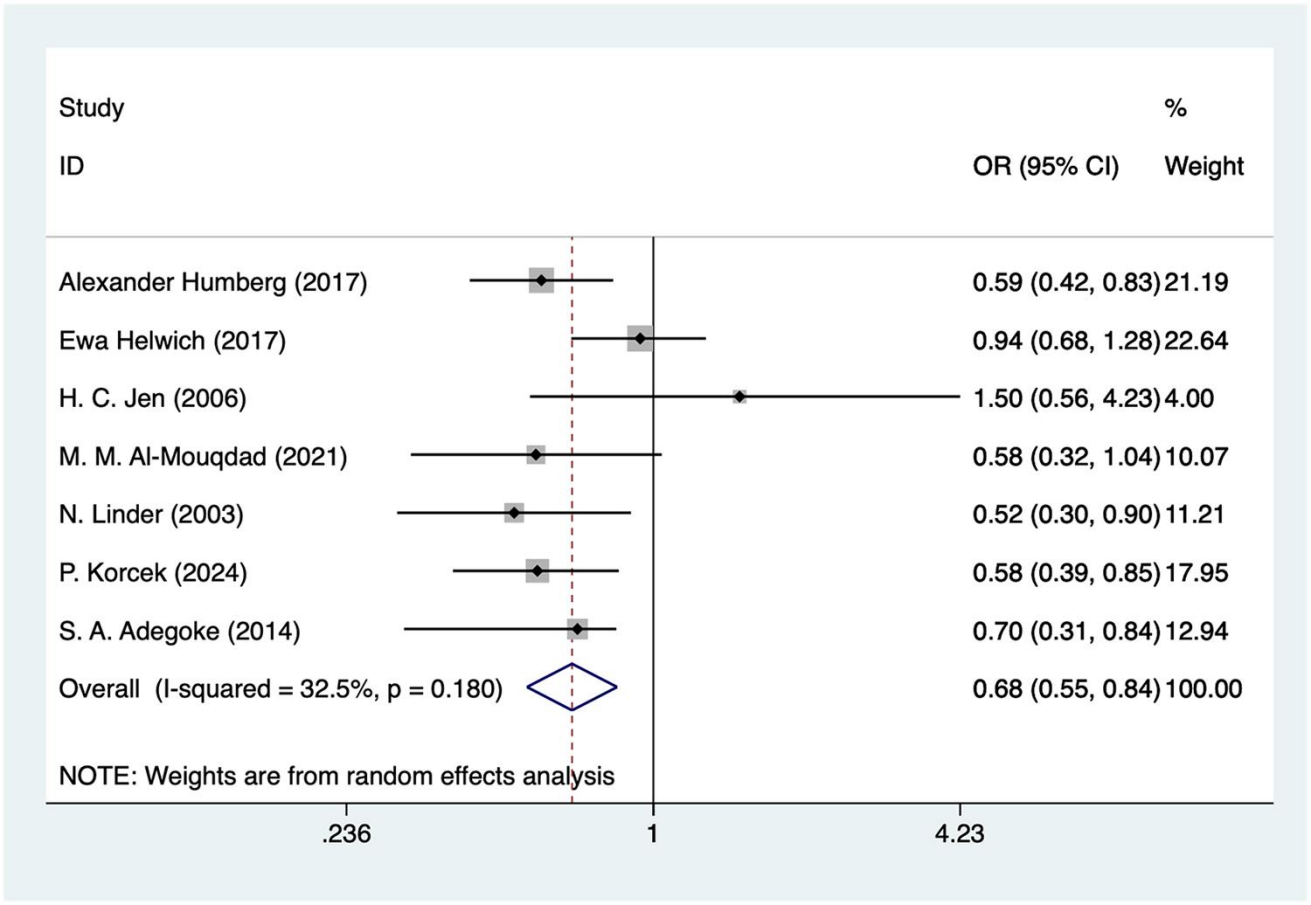
Forest plot of antenatal corticosteroids meta-analysis.

Seven studies mentioned antenatal corticosteroids, and the pooled results (Figure 4) indicated that antenatal was associated with a decreased risk of IVH in VLBW infants [OR = 0.68, 95%CI (0.55, 0.84), *P* = 0.001].

### Vaginal delivery

**Figure 5:**
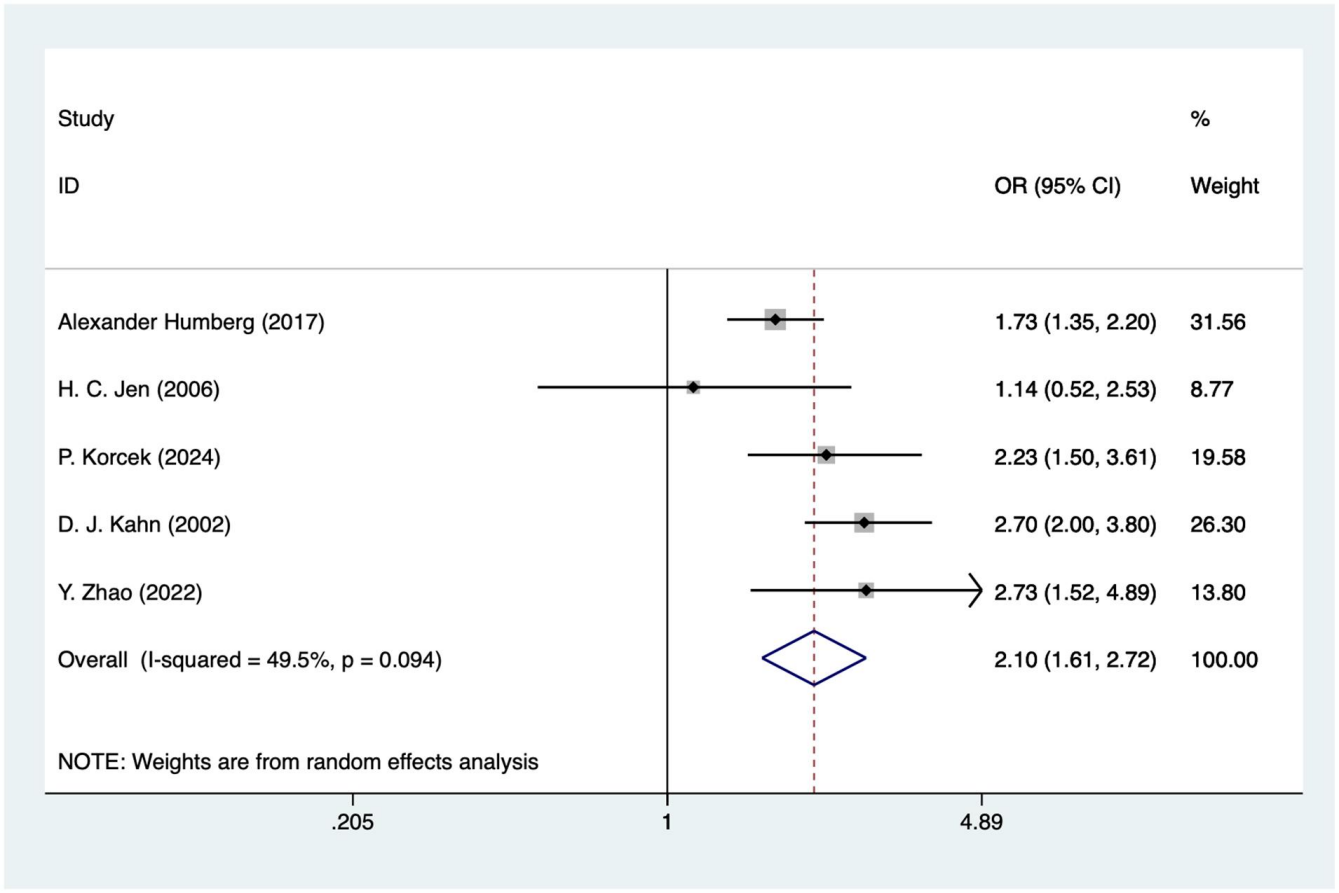
Forest plot of vaginal delivery meta-analysis.

Five studies mentioned vaginal delivery, and the pooled results (Figure 5) indicated that vaginal delivery was associated with an increased risk of IVH in VLBW infants [OR = 2.10, 95%CI (1.61, 2.72), *P* = 0.002].

### Neonatal thrombocytopenia

**Figure 6:**
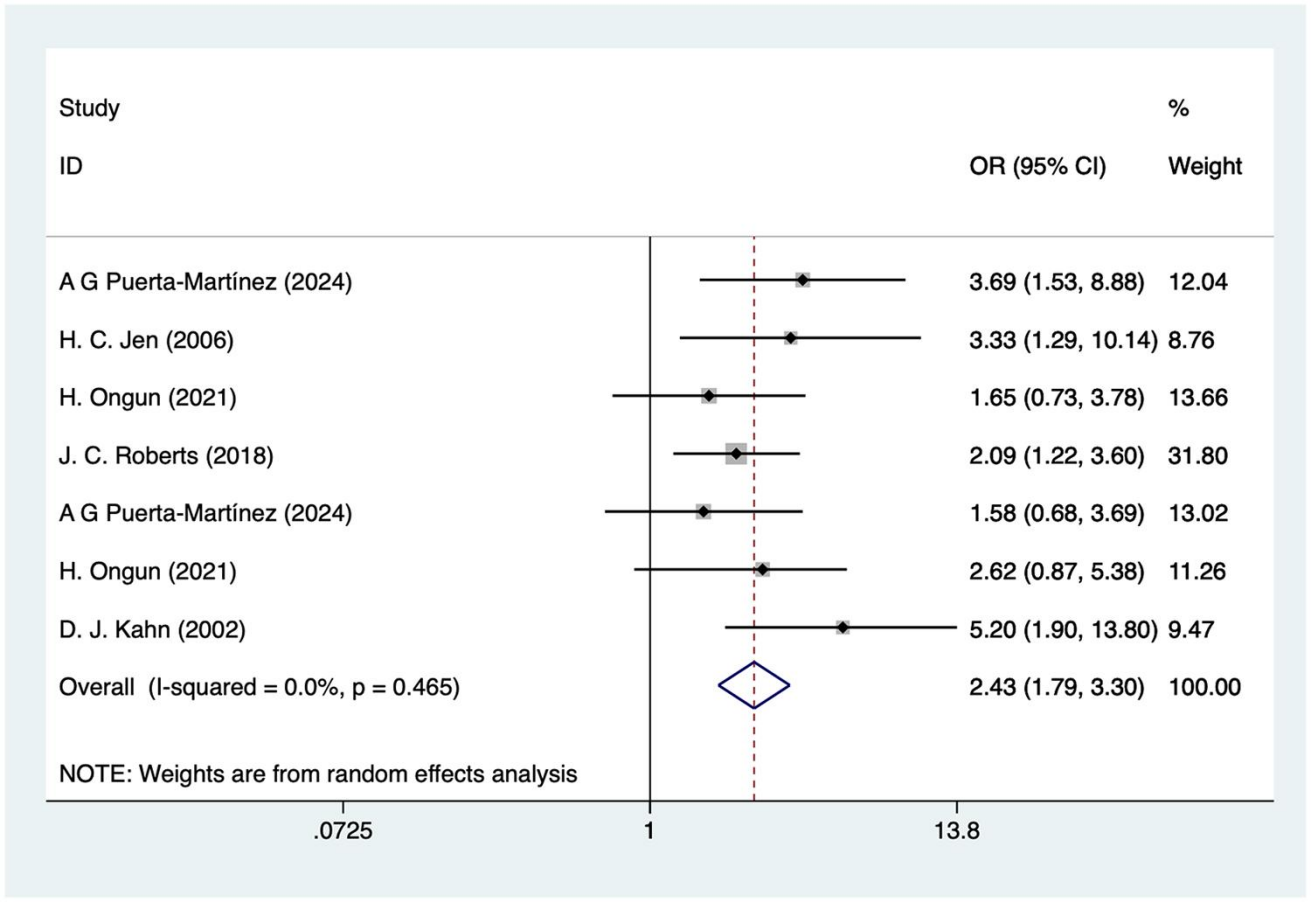
Forest plot of neonatal thrombocytopenia meta-analysis.

Five studies mentioned neonatal thrombocytopenia, and the pooled results (Figure 6) indicated that neonatal thrombocytopenia was associated with an increased risk of IVH in VLBW infants [OR = 2.43, 95%CI (1.79, 3.30), *P* = 0.01].

### Pulmonary hemorrhage

**Figure 7:**
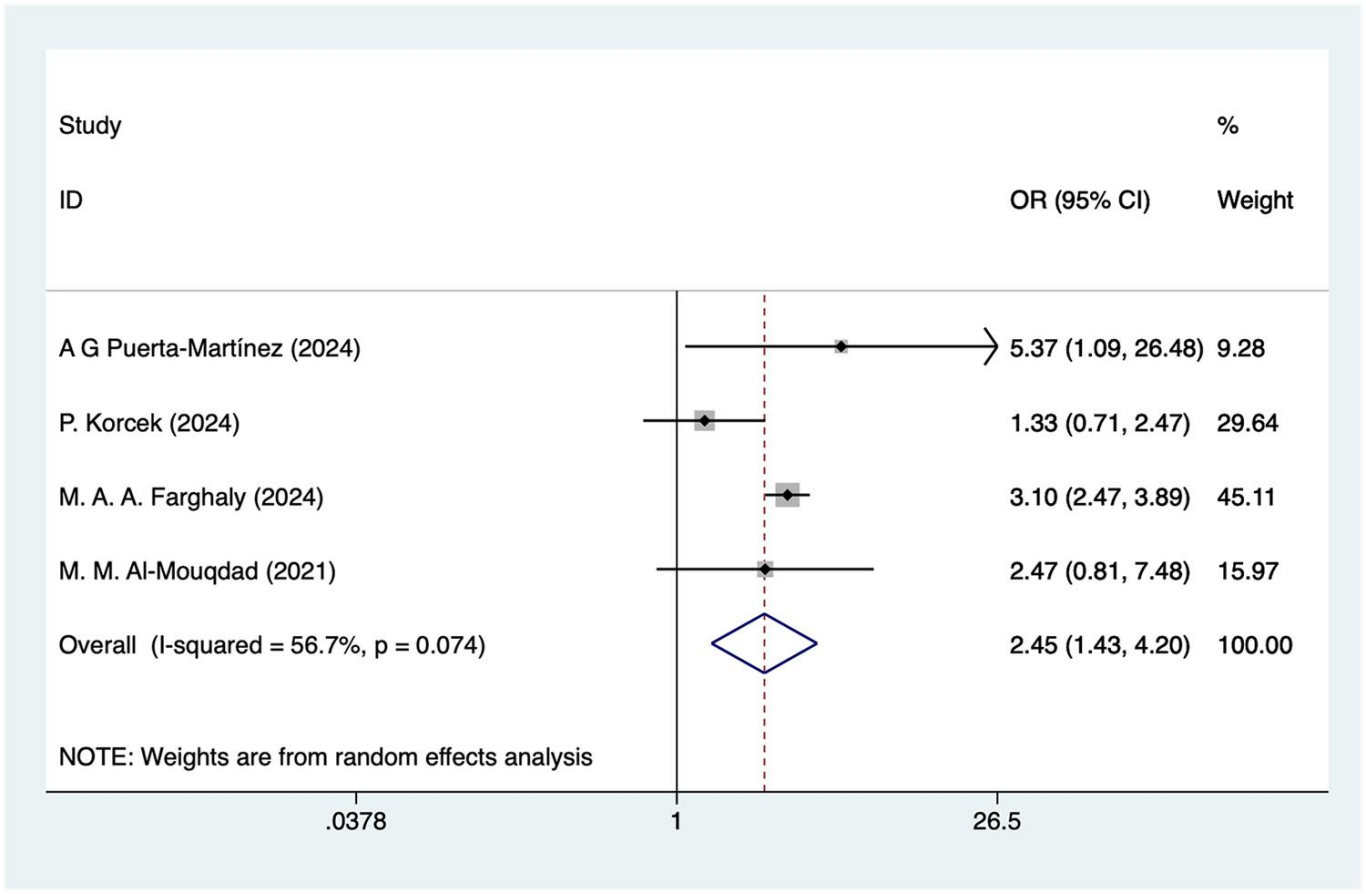
Forest plot of pulmonary hemorrhage meta-analysis.

Four studies mentioned pulmonary hemorrhage, and the pooled results (Figure 7) indicated that pulmonary hemorrhage was associated with an increased risk of IVH in VLBW infants [OR = 2.45, 95%CI (1.43, 4.20), *P* = 0.008]. Due to the high heterogeneity, sensitivity analysis was performed using literature-by-exclusion, and the results of the analysis (Supplementary Figure S2) suggested that the sensitivity was low and that the results of the analysis were not affected by an individual study.

### Mechanical

**Figure 8:**
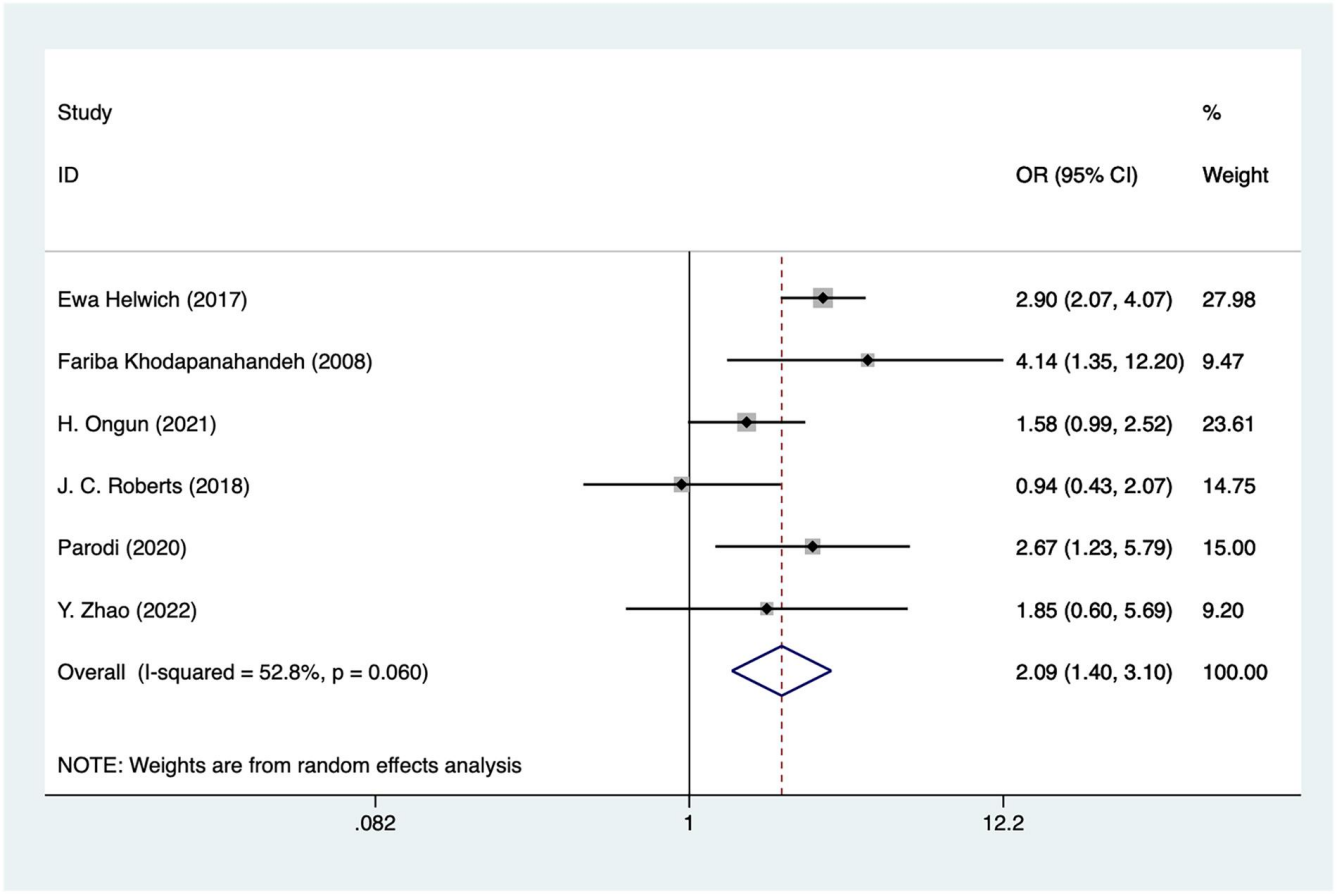
Forest plot of mechanical meta-analysis.

Six studies mentioned mechanical, and the pooled results (Figure 8) indicated that mechanical was associated with an increased risk of IVH in VLBW infants [OR = 2.09, 95%CI (1.40, 3.10), *P* = 0.02]. Due to the high heterogeneity, sensitivity analysis was performed using literature-by-exclusion, and the results of the analysis (Supplementary Figure S3) suggested that the sensitivity was low and that the results of the analysis were not affected by an individual study.

### Sepsis

**Figure 9:**
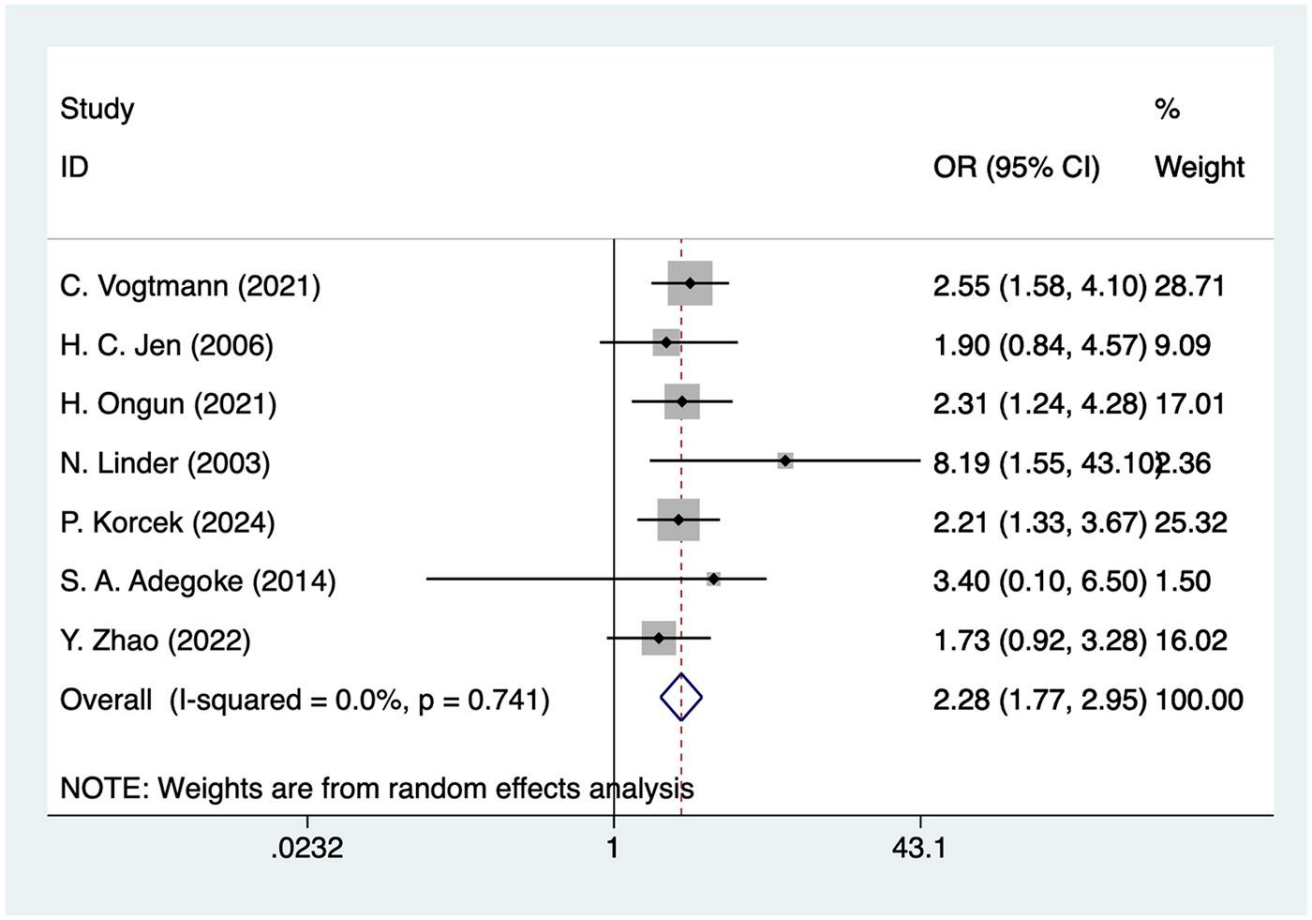
Forest plot of sepsis meta-analysis.

Seven studies mentioned sepsis, and the pooled results (Figure 9) indicated that sepsis was associated with an increased risk of IVH in VLBW infants [OR = 2.28, 95%CI (1.77, 2.95), *P* = 0.001].

### Publication bias

Funnel plot and Egger's test were used to detect publication bias in the current study and the results are shown in (Supplementary Figures S4–S11), where hypotension (*P* = 0.35), patent ductus arteriosus (*P* = 0. 338), antenatal corticosteroids (*P* = 0.930), vaginal delivery (*P* = 0.945), neonatal thrombocytopenia (*P* = 0.242), pulmonary hemorrhage (*P* = 0.689), mechanical (*P* = 0.642), and sepsis (*P* = 0.265) suggesting a low likelihood of publication bias.

## Discussion

This study identified multiple risk factors associated with IVH, including hypotension, patent ductus arteriosus, antenatal corticosteroids, vaginal delivery, neonatal thrombocytopenia, pulmonary hemorrhage, mechanical ventilation and sepsis. These findings provide valuable insights into IVH management and prevention in VLBW.

### Hypotension and IVH in VLBW

Hypotension is a significant risk factor for the development of IVH in VLBW. The ratio (OR) of the meta-analysis was 3.64, suggesting that hypotension almost quadrupled the risk of developing IVH. This result is consistent with previous studies that hypotension is strongly associated with the occurrence of intraventricular hemorrhage (43). Hypotension is often accompanied by poor cerebral perfusion, which increases the likelihood of hemorrhage in the fragile fetal matrix. Studies have shown that in preterm infants, due to incomplete development of the cerebral vasculature, hypotension leads to reduced cerebral blood flow, particularly in the periventricular region, which predisposes to the development of hemorrhage (44, 45). Similar studies have found that low blood pressure is positively associated with the development of IVH [Song et al., (46)].

### Patent ductus arteriosus and IVH in VLBW

The association of patent ductus arteriosus with the development of IVH in VLBW has been widely reported in the literature. Hemodynamically significant PDA (hsPDA), characterized by a large ductal diameter, increased left-to-right shunt, and evidence of systemic hypoperfusion, appears to confer a higher risk of IVH (47). Moreover, differences in PDA management may further influence the risk of IVH. Infants with non-operated or conservatively managed PDA, particularly those with persistent hsPDA, may experience prolonged exposure to cerebral hypoperfusion, hypoxemia, and hypercapnia, which further increase cerebrovascular vulnerability (48). In contrast, early pharmacological or surgical closure of hsPDA may stabilize systemic and cerebral hemodynamics and potentially reduce the risk of IVH, although evidence remains inconsistent across studies. Additionally, persistent hypoxemia and hypercapnia in the absence of effective PDA closure may elevate cerebral venous pressure and disrupt fragile germinal matrix vessels, thereby increasing the likelihood of IVH (49).

### Antenatal corticosteroids and IVH in VLBW

The meta-analysis of this study confirmed this protective effect with an OR of 0.69, suggesting that antenatal use of corticosteroids significantly reduces the risk of IVH in VLBW. Antenatal corticosteroids exert a protective effect by enhancing lung maturation, reducing pulmonary complications and improving neonatal stability (50). timing and completeness of treatment. Evidence suggests that administration of a complete course of antenatal corticosteroids, typically within 7 days before preterm delivery, provides the greatest reduction in IVH risk. Studies have shown that corticosteroids can reduce the risk of IVH by inducing fetal lung maturation, improving oxygen exchange, and reducing the incidence of hypoxia, thereby reducing cerebrovascular pressure (51, 52). Moreover, exposure to antenatal corticosteroids 24 h to 7 days before birth has been associated with optimal neonatal outcomes, whereas incomplete courses or administration far in advance of delivery may confer a reduced protective effect (53). Repeated or “rescue” courses remain controversial, with inconsistent evidence regarding additional benefits for IVH prevention.

### Vaginal delivery and IVH in VLBW

The meta-analysis of this study found that vaginal delivery was indeed a risk factor for IVH with an OR of 2.06. This result is consistent with existing studies that the acute abdominal pressure and the pulling of the fetal head during delivery may lead to rupture of blood vessels in the brain and increase the likelihood of IVH (54). Notably, the observed association may be influenced by confounding factors such as gestational age, fetal presentation, emergency vs. elective cesarean section, and underlying maternal or fetal conditions. Therefore, although our findings suggest an increased risk of IVH associated with vaginal delivery in VLBW infants, the contradictory results across studies highlight the need for well-designed prospective studies that adequately control for these confounding factors before definitive conclusions can be drawn.

### Neonatal thrombocytopenia and IVH in VLBW

Neonatal thrombocytopenia was another risk factor significantly associated with IVH with an OR of 2.43. Low platelet levels may make neonates more susceptible to hemorrhage, including cerebral hemorrhage (30). Thrombocytopenia is common in preterm infants, especially premature, low-birth-weight neonates. Platelets play an important role in blood clotting, and thrombocytopenia leads to an increased risk of blood vessel rupture (55).

### Pulmonary hemorrhage and IVH in VLBW

Pulmonary hemorrhage is a common complication of VLBW and may result from pulmonary vascular immaturity and mechanical ventilation. In the present study, pulmonary hemorrhage was found to be a risk factor for IVH with an OR of 2.45. Pulmonary hemorrhage is often accompanied by severe respiratory distress and poor oxygenation, leading to hemodynamic instability and further increasing the risk of cerebral hemorrhage (56). Pulmonary hemorrhage may affect cardiopulmonary function in preterm infants, leading to changes in systemic blood flow, which in turn affects cerebrovascular perfusion (57).

### Mechanical ventilation and IVH in VLBW

Mechanical ventilation is a commonly used treatment for VLBW, especially for pulmonary diseases such as neonatal respiratory distress syndrome (RDS), but it is strongly associated with the risk of IVH. In the present study (58), mechanical ventilation was found to be a risk factor for IVH with an OR of 2.09. Mechanical ventilation may lead to changes in cerebrovascular pressure by altering thoracic pressure, affecting cerebral blood flow and oxygenation status, and increasing the risk of developing IVH (59). Ventilation duration, particularly during the first few days of life, is associated with a higher incidence of IVH in VLBW infants, whereas early weaning or the use of non-invasive respiratory support may mitigate this risk (60). However, ventilation duration is often closely linked to disease severity, and residual confounding cannot be fully excluded.

### Sepsis and IVH in VLBW

Sepsis is a common serious infection in preterm infants and is a significant risk factor for IVH with an OR of 2.28. Sepsis increases the risk of hemorrhage by inducing a systemic inflammatory response and hemodynamic instability that may affect cerebral perfusion (61). The systemic inflammatory response triggered by sepsis leads to increased vascular permeability and impaired blood coagulation, further contributing to the development of cerebral hemorrhage.

### Comparative interpretation of effect sizes

The strength of association between different risk factors and intraventricular hemorrhage (IVH) in very low birth weight (VLBW) infants varied substantially. Hypotension showed the strongest association (OR = 3.64), highlighting the role of impaired systemic and cerebral perfusion in IVH. Other risk factors, including pulmonary hemorrhage (OR = 2.45), neonatal thrombocytopenia (OR = 2.43), sepsis (OR = 2.28), mechanical ventilation (OR = 2.09), and vaginal delivery (OR = 2.06), demonstrated moderate-to-strong associations, reflecting overlapping mechanisms such as hemodynamic instability, inflammation, coagulation dysfunction, and illness severity. Patent ductus arteriosus (OR = 1.86) showed a smaller but significant effect size, likely due to variations in severity and management. Antenatal corticosteroid exposure was the only protective factor (OR = 0.69), reinforcing its role in IVH prevention. Larger effect sizes indicate direct pathophysiological drivers, while moderate ones may represent mediators or markers in a complex causal network. These results suggest that early hemodynamic stabilization and targeted preventive strategies are key to reducing IVH risk.

### Clinical significance

This review identifies clinically relevant risk factors for IVH in VLBW infants, including hypotension, patent ductus arteriosus, antenatal corticosteroids, vaginal delivery, thrombocytopenia, pulmonary hemorrhage, mechanical ventilation, and sepsis. Larger effect sizes, particularly for hypotension and PDA, emphasize the need for early risk stratification, hemodynamic monitoring, and stabilization. Conditions such as thrombocytopenia, pulmonary hemorrhage, and sepsis highlight the importance of integrated supportive care to prevent cerebrovascular instability. Antenatal corticosteroids remain crucial for perinatal care, especially in preterm deliveries. The association with vaginal delivery suggests it should be considered in an individualized risk-benefit framework. Overall, these findings advocate for a multifactorial approach to IVH prevention, emphasizing early identification, optimized management, and personalized clinical decision-making.

### Strengths and limitations

The study's strength lies in its comprehensive analysis of 21 high-quality studies from multiple regions, ensuring broad applicability. It identifies key clinical interventions and risk factors, with high-quality studies and strong bias assessment. However, limitations include heterogeneity in pooled analyses, particularly for hypotension and pulmonary hemorrhage, which may have reduced precision. The observational design of most studies limits causal inference and missing or inconsistent data could have introduced bias. The exclusion of non-English studies may have introduced publication and language bias. Subgroup analyses based on extreme prematurity or other strata were not possible due to inconsistent reporting, limiting refined risk assessments. Regional differences in clinical practice may further limit generalizability, especially in low-resource settings.

## Conclusion

The findings of this systematic review and meta-analysis indicate that significant risk factors for IVH in VLBW infants include hypotension, PDA, antenatal corticosteroid use, vaginal delivery, neonatal thrombocytopenia, pulmonary hemorrhage, mechanical ventilation, and sepsis. Whilst these factors exhibit strong clinical associations with IVH, it is noteworthy that current understanding of IVH Patho mechanisms—such as basal vascular fragility, cerebral autoregulatory dysfunction, inflammatory processes, and hemodynamic instability—primarily derives from preclinical studies. Although clinical evidence remains crucial in elucidating these mechanisms, integrating findings from preclinical research will contribute to a more comprehensive understanding of IVH pathogenesis and inform early intervention strategies. Consequently, future research should strive to bridge the gap between clinical observations and preclinical studies, such as animal models, to better inform preventive measures.

## Data Availability

The original contributions presented in the study are included in the article/Supplementary Material, further inquiries can be directed to the corresponding author.
