# Supplementary material for: Risk factors for intraventricular hemorrhage in very low birth weight infants: a systematic review and meta-analysis
Source: Front Pediatr. 2026 Jan 26;13:1728632. doi: 10.3389/fped.2025.1728632 (PMC12883645; doi:10.3389/fped.2025.1728632)
Supplement: Supplementary file 1 [file Datasheet1.docx]

Table S1 search strategy

(((Infant, Very Low Birth Weight[MeSH Terms]) OR (((((((Infant, Very Low Birth Weight[Title/Abstract]) OR (Very-Low-Birth-Weight Infant[Title/Abstract])) OR (Infants, Very-Low-Birth-Weight[Title/Abstract])) OR (Infant, Very-Low-Birth-Weight[Title/Abstract])) OR (Very Low Birth Weight Infant[Title/Abstract])) OR (Very-Low-Birth-Weight Infants[Title/Abstract])) OR (Very Low Birth Weight[Title/Abstract]))) AND ((Cerebral Intraventricular Hemorrhage[MeSH Terms]) OR ((((((((((Cerebral Intraventricular Hemorrhage[Title/Abstract]) OR (Cerebral Intraventricular Hemorrhages[Title/Abstract])) OR (Hemorrhage, Cerebral Intraventricular[Title/Abstract])) OR (Intraventricular Hemorrhage, Cerebral[Title/Abstract])) OR (Cerebral Intraventricular Haemorrhage[Title/Abstract])) OR (Cerebral Intraventricular Haemorrhages[Title/Abstract])) OR (Haemorrhage, Cerebral Intraventricular[Title/Abstract])) OR (Intraventricular Haemorrhage, Cerebral[Title/Abstract])) OR (Intraventricular Haemorrhages, Cerebral[Title/Abstract])) OR (Intraventricular hemorrhage[Title/Abstract])))) AND ((Risk Factors[MeSH Terms]) OR (((((((((((((((Risk Factors[Title/Abstract]) OR (Factor, Risk[Title/Abstract])) OR (Risk Factor[Title/Abstract])) OR (Population at Risk[Title/Abstract])) OR (Populations at Risk[Title/Abstract])) OR (Risk Scores[Title/Abstract])) OR (Risk Score[Title/Abstract])) OR (Score, Risk[Title/Abstract])) OR (Risk Factor Scores[Title/Abstract])) OR (Risk Factor Score[Title/Abstract])) OR (Score, Risk Facto[Title/Abstract])) OR (Health Correlates[Title/Abstract])) OR (Correlates, Health[Title/Abstract])) OR (Social Risk Factors[Title/Abstract])) OR (Factor, Social Risk[Title/Abstract])))


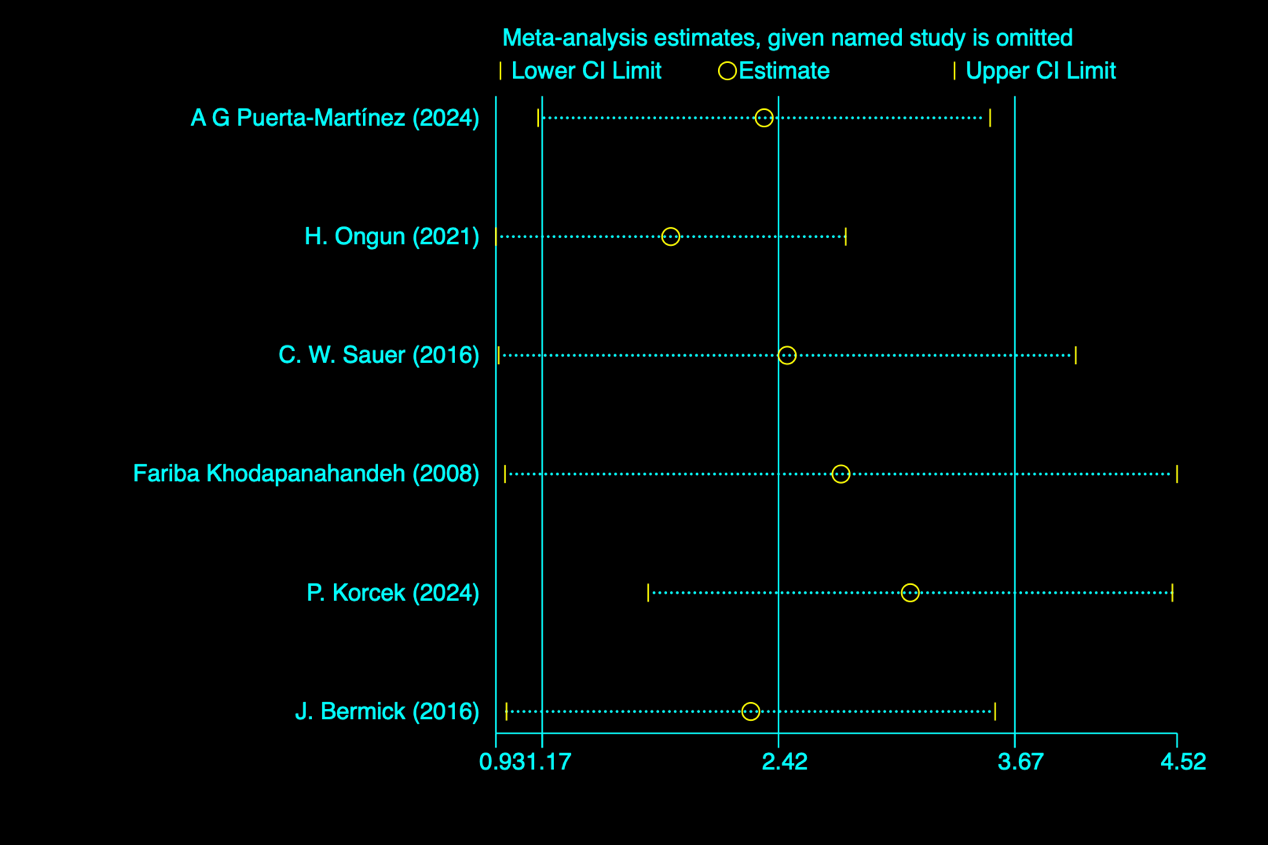


Figure S1 Sensitivity analysis of Hypotension


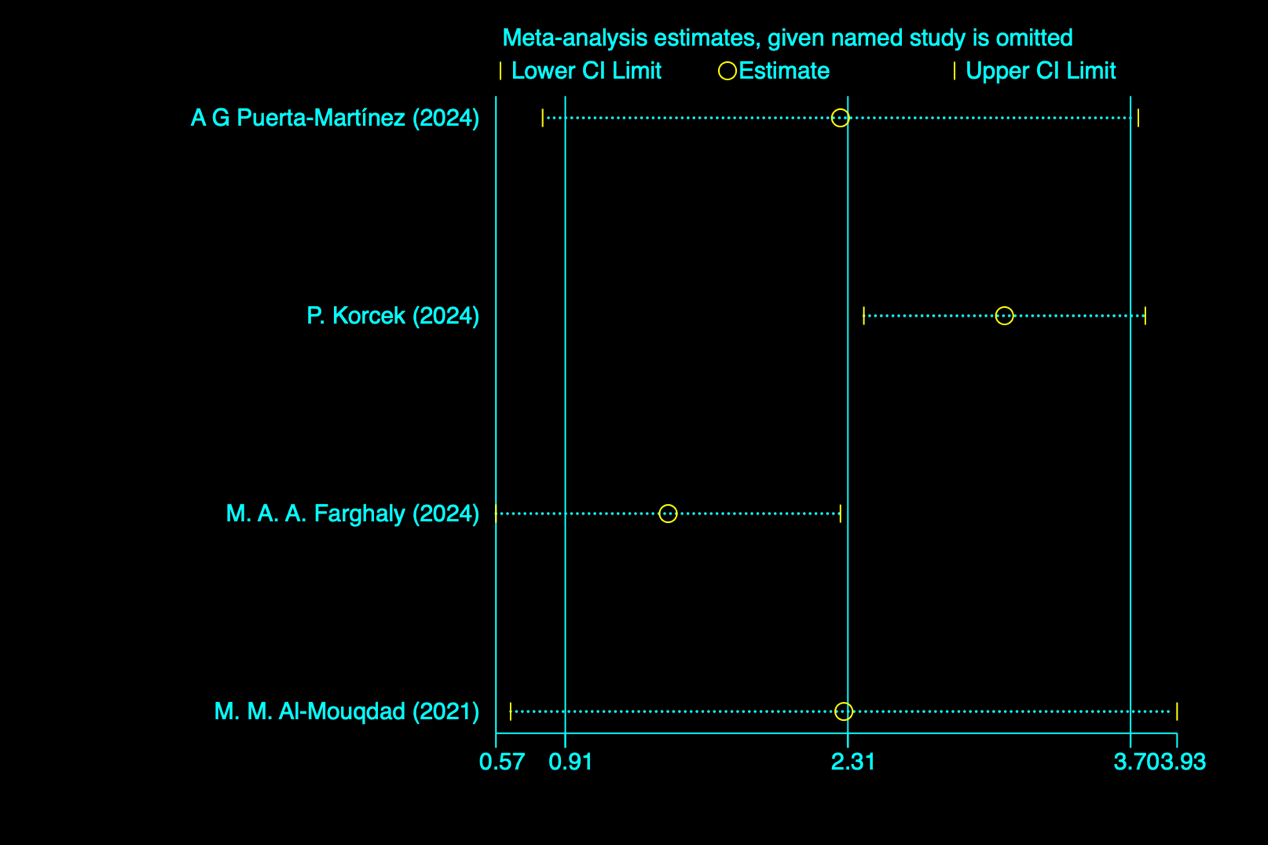


Figure S2 Sensitivity analysis of Pulmonary hemorrhage


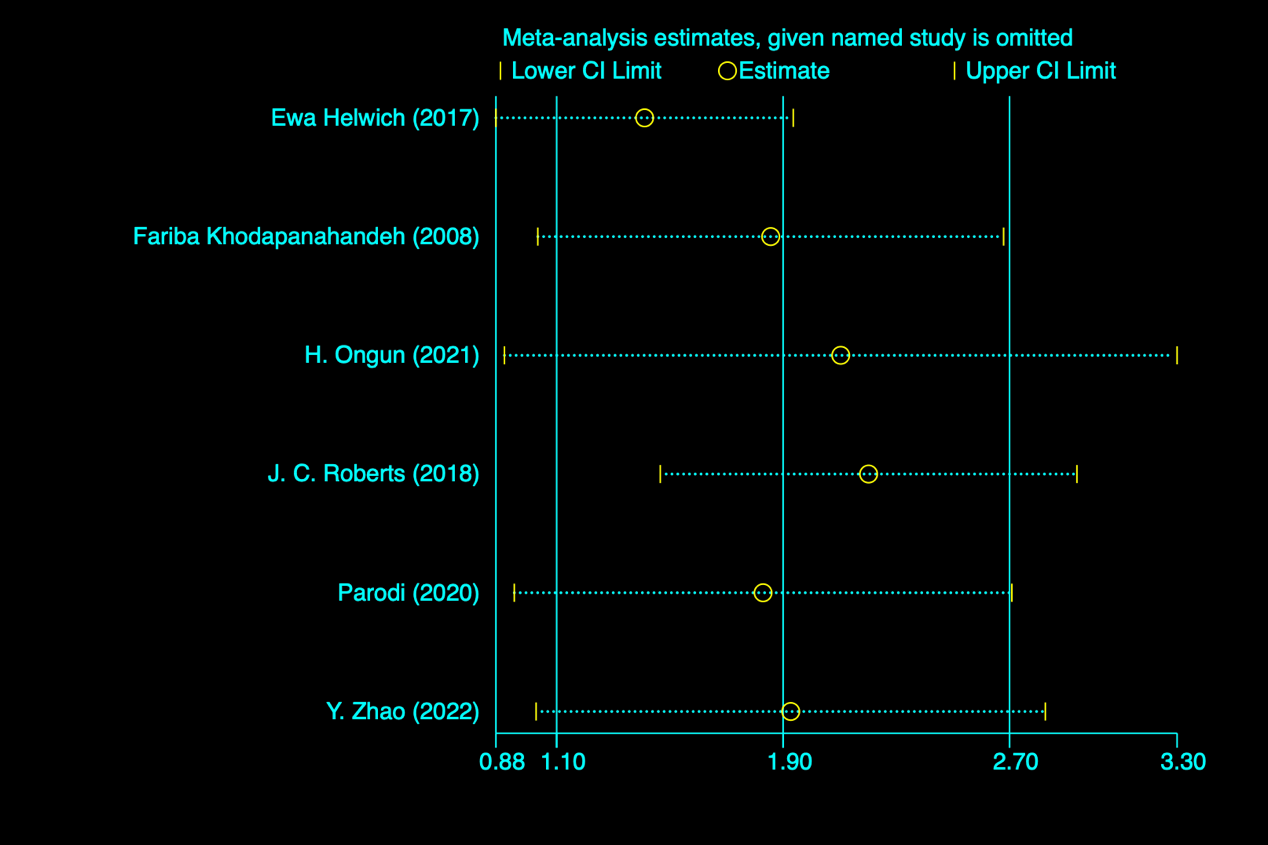


Figure S3 Mechanical Sensitivity Analysis


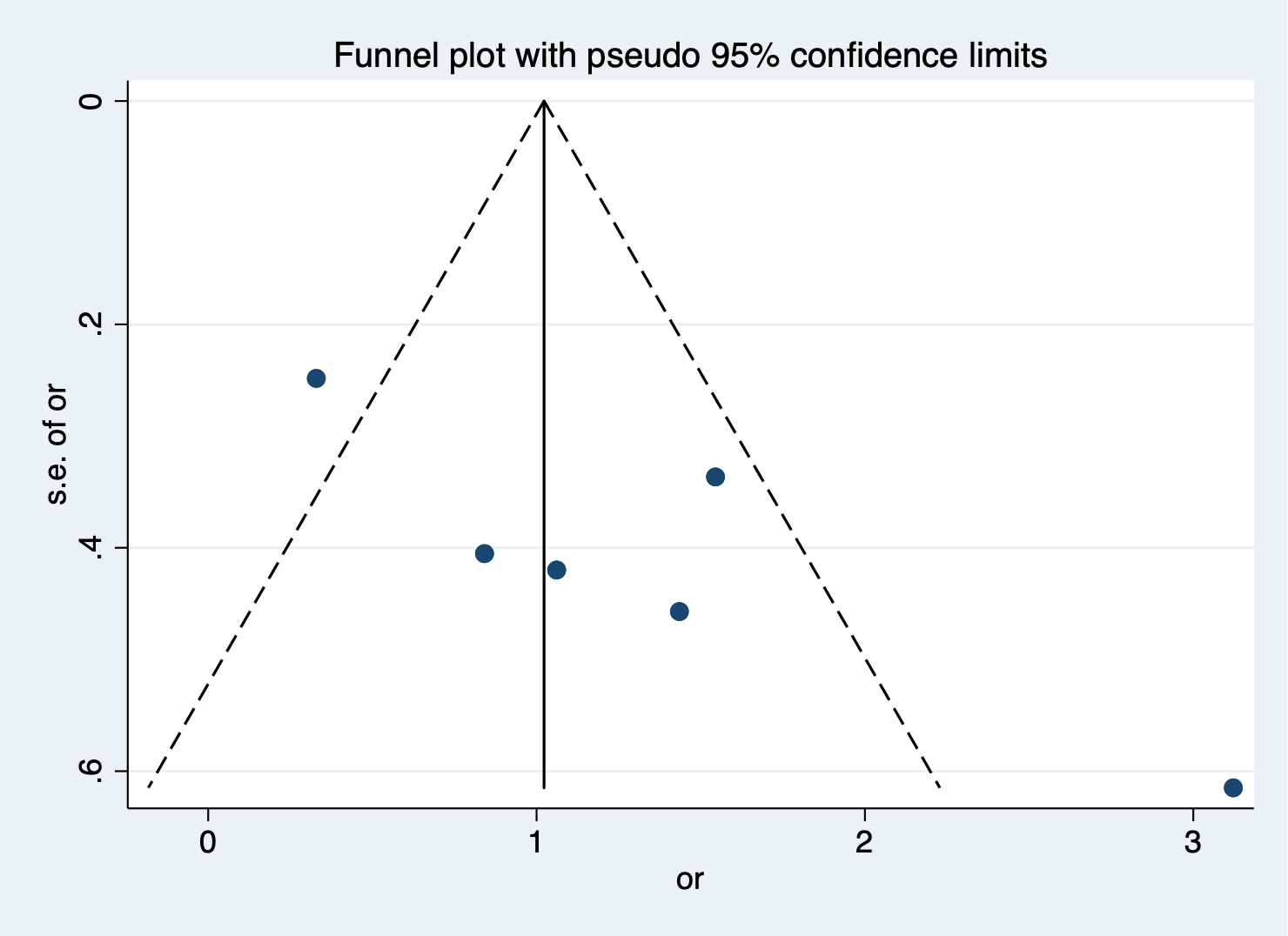


Figure S4 Funnel plot of Hypotension meta-analysis


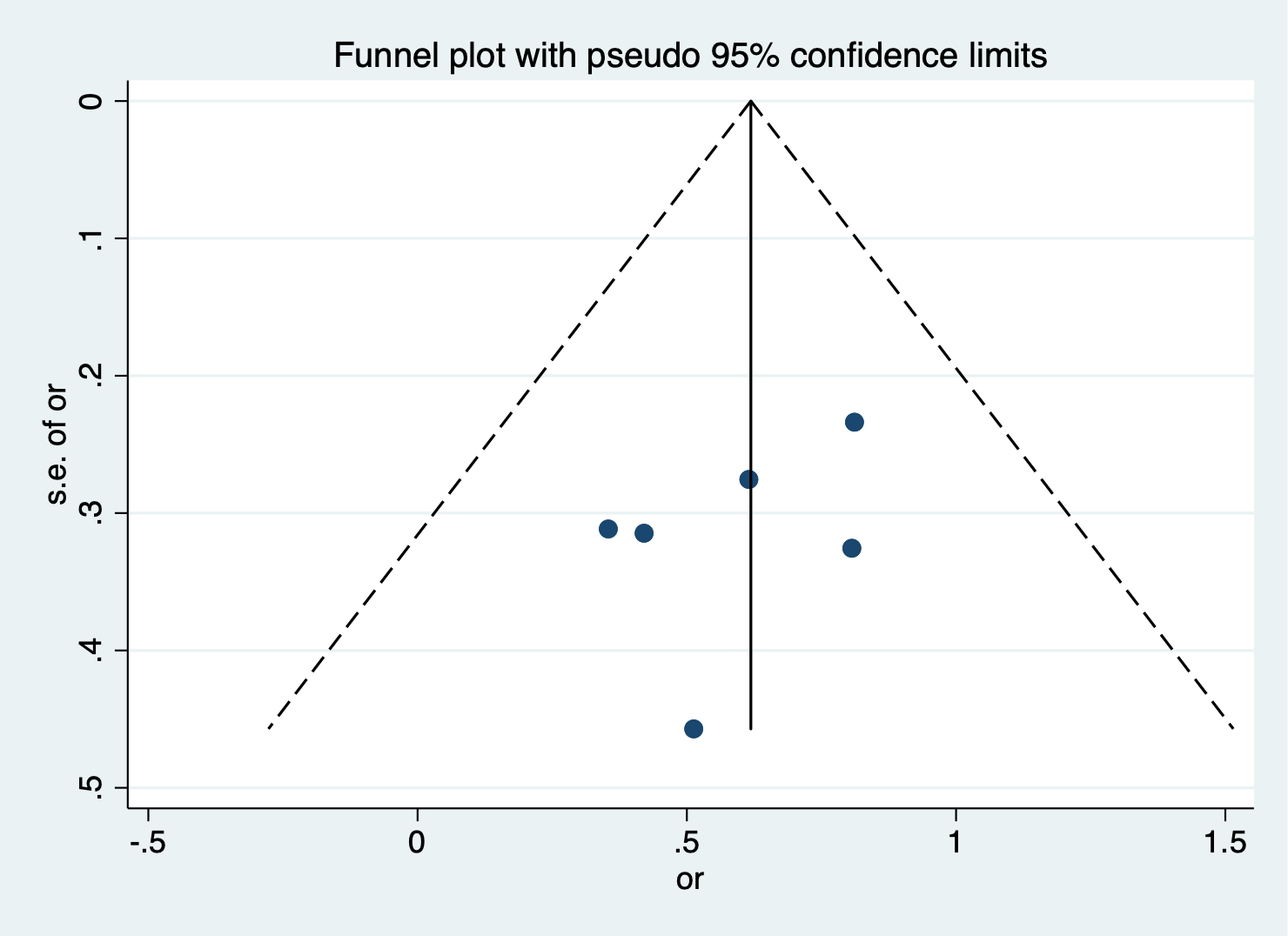


Figure S5 Funnel plot of patent ductus arteriosus meta-analysis


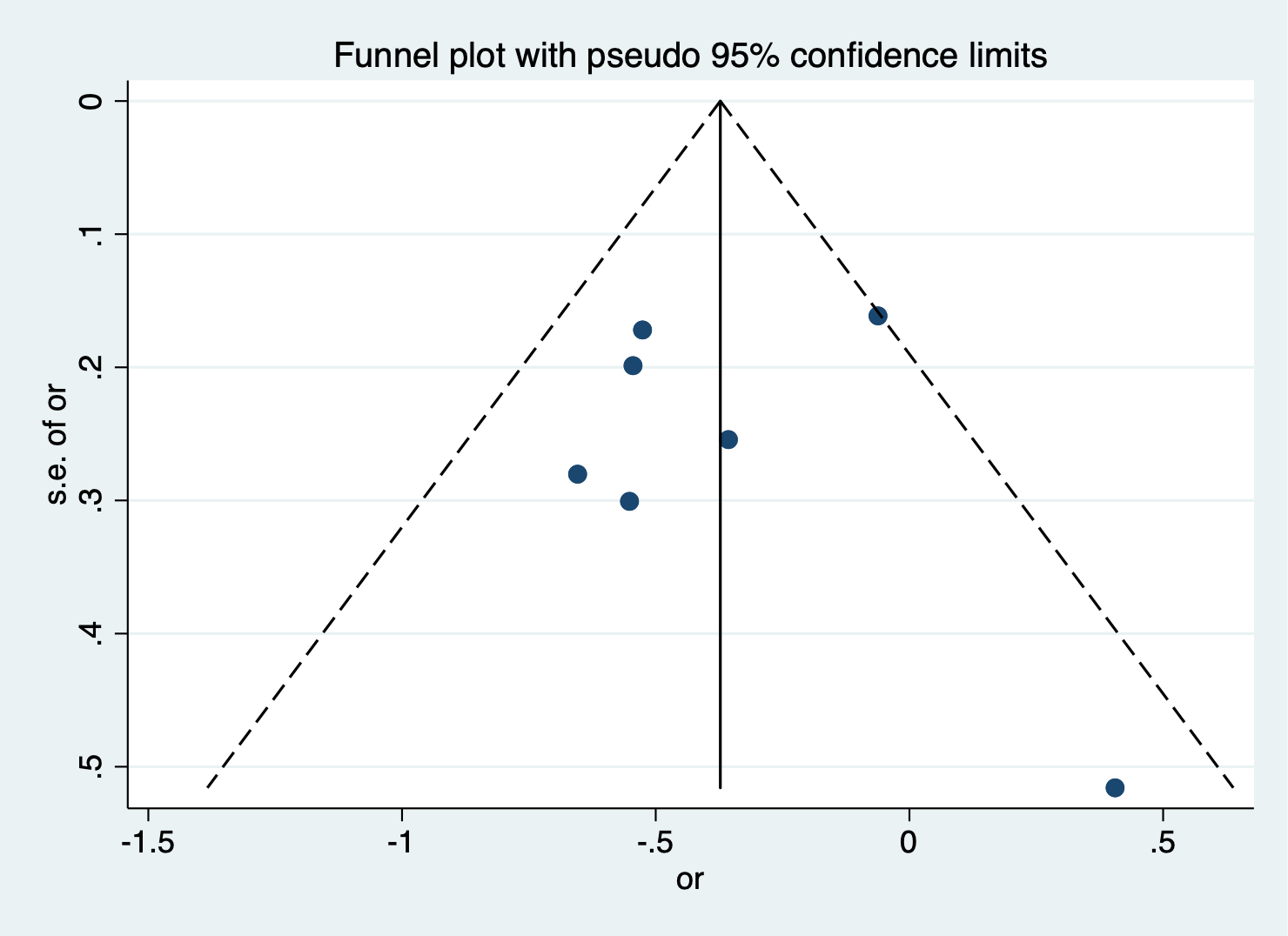


Figure S6 Funnel plot of antenatal corticosteroids meta-analysis


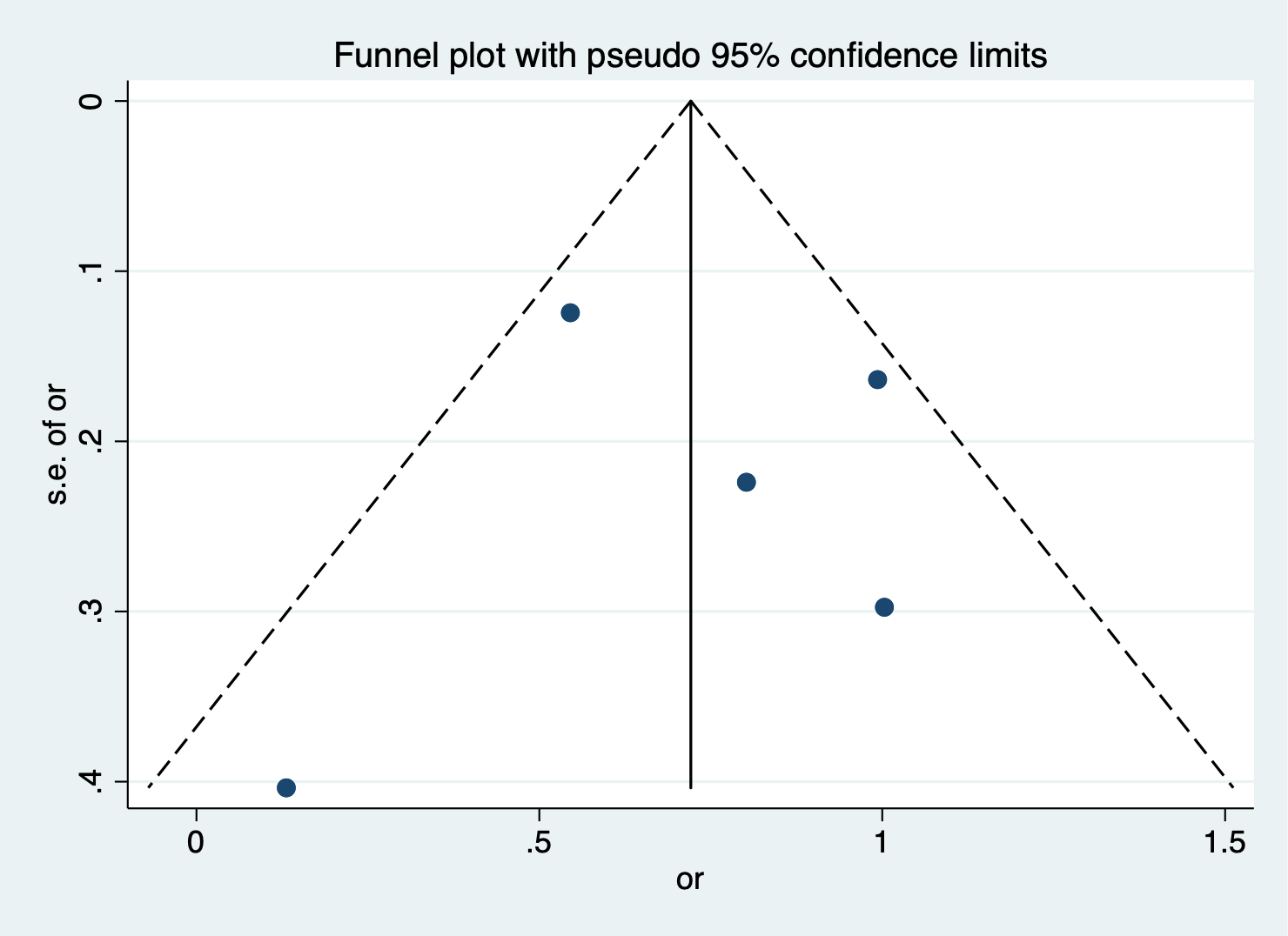


Figure S7 Funnel plot of vaginal delivery meta-analysis


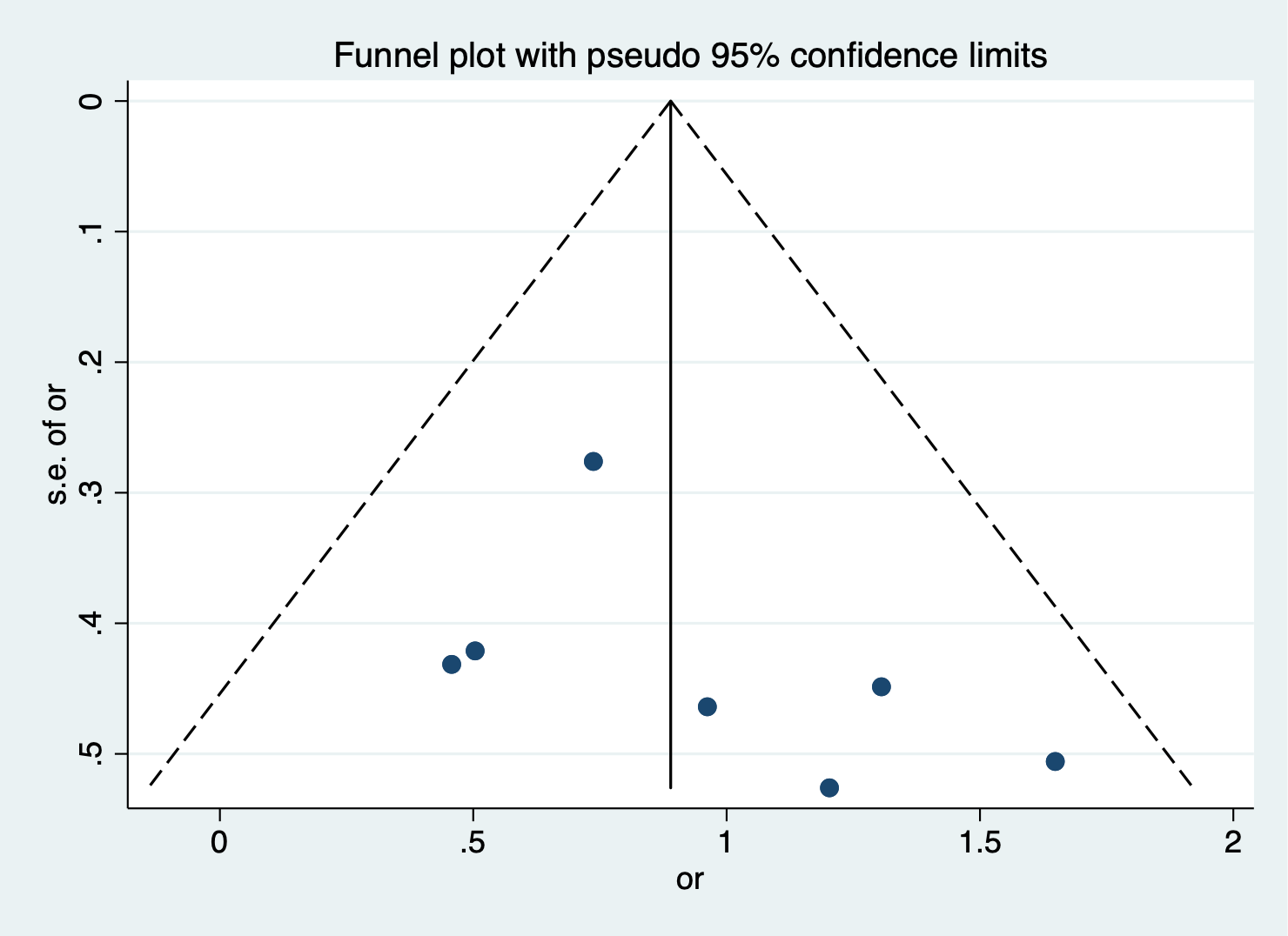


Figure S8 Funnel plot of neonatal thrombocytopenia meta-analysis


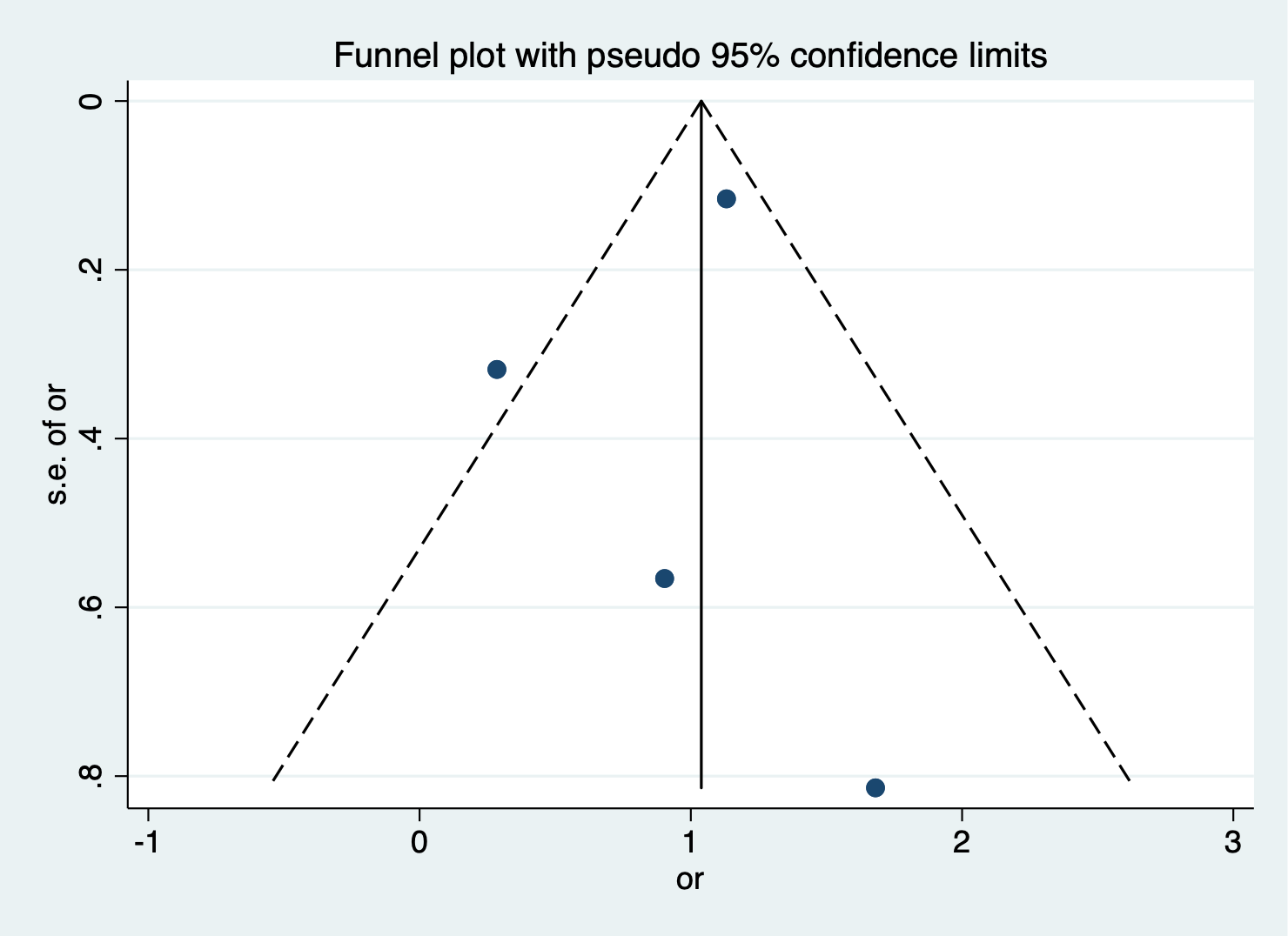


Figure S9 Funnel plot of pulmonary hemorrhage meta-analysis


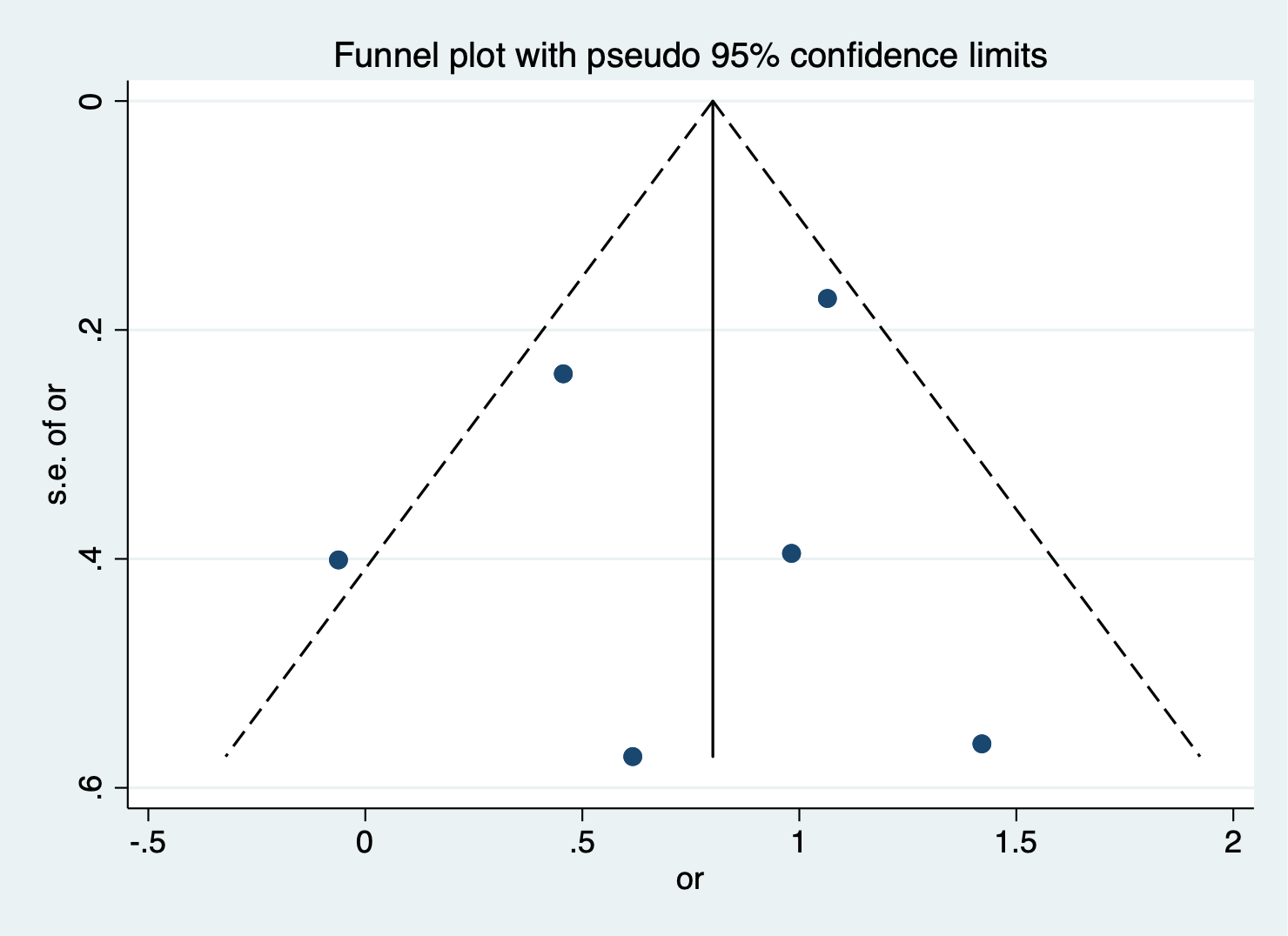


Figure S10 Funnel plot of mechanical meta-analysis


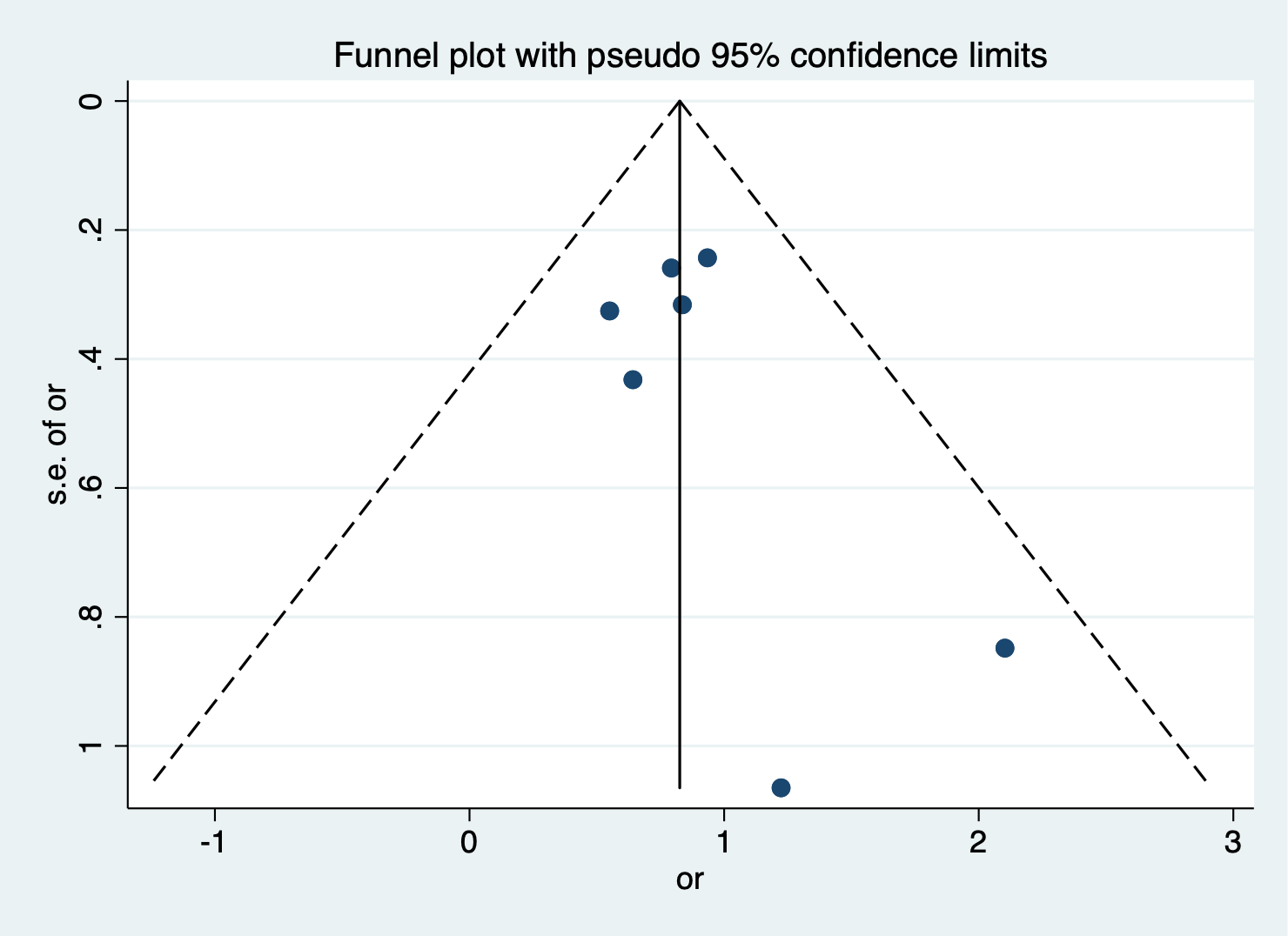


Figure S11 Funnel plot of sepsis meta-analysis
